# Adherence and Association of Digital Proximity Tracing App Notifications With Earlier Time to Quarantine: Results From the Zurich SARS-CoV-2 Cohort Study

**DOI:** 10.3389/ijph.2021.1603992

**Published:** 2021-08-16

**Authors:** Tala Ballouz, Dominik Menges, Hélène E. Aschmann, Anja Domenghino, Jan S. Fehr, Milo A. Puhan, Viktor von Wyl

**Affiliations:** ^1^ Epidemiology, Biostatistics and Prevention Institute, University of Zurich, Zürich, Switzerland; ^2^ Department of Infectious Diseases and Hospital Epidemiology, University Hospital Zürich, Zürich, Switzerland; ^3^ Department of Visceral and Transplantation Surgery, University Hospital Zürich, Zürich, Switzerland; ^4^ Institute for Implementation Science in Health Care, University of Zurich, Zürich, Switzerland

**Keywords:** SARS-CoV-2, DPT, digital proximity tracing, contact tracing, quarantine, close contacts

## Abstract

**Objectives:** We aimed to evaluate the effectiveness of the SwissCovid digital proximity tracing (DPT) app in notifying exposed individuals and prompting them to quarantine earlier compared to individuals notified only by manual contact tracing (MCT).

**Methods:** A population-based sample of cases and close contacts from the Zurich SARS-CoV-2 Cohort was surveyed regarding SwissCovid app use and SARS-CoV-2 exposure. We descriptively analyzed app adherence and effectiveness, and evaluated its effects on the time between exposure and quarantine among contacts using stratified multivariable time-to-event analyses.

**Results:** We included 393 SARS-CoV-2 infected cases and 261 close contacts. 62% of cases reported using SwissCovid and among those, 88% received and uploaded a notification code. 71% of close contacts were app users, of which 38% received a warning. Non-household contacts notified by SwissCovid started quarantine 1 day earlier and were more likely to quarantine earlier than those not warned by the app (HR 1.53, 95% CI 1.15–2.03).

**Conclusion:** These findings provide evidence that DPT may reach exposed contacts faster than MCT, with earlier quarantine and potential interruption of SARS-CoV-2 transmission chains.

## Introduction

Contact tracing is a crucial public health measure for controlling the spread of severe acute respiratory syndrome coronavirus 2 (SARS-CoV-2) [1, 2]. Traditionally, contact tracing involves interviewing all infected individuals (index cases) to systematically identify their close contacts. This aims at interrupting viral transmission chains by referring these close contacts to quarantine and SARS-CoV-2 testing [2–4]. However, such manual contact tracing (MCT) has inherent time delays, is resource intensive, and is limited by imperfect recall of encounters, especially those occurring briefly or by chance. Given the rapid SARS-CoV-2 transmission and the high proportion of asymptomatic cases, MCT alone is thus unlikely to be sufficiently effective [5, 6]. Digital proximity tracing (DPT) has been developed as a scalable complementary method to identify transmission chains that are likely to be missed or identified late by MCT [7, 8].

DPT applications register proximity encounters between individuals using the app with the aim to identify individuals that may have been exposed to an index case. Different technologies and architectures for DPT exist, one of which is decentralized, privacy-preserving proximity tracing (DP-3T) using Bluetooth Low Energy signals [9, 10]. The Swiss DPT app, «SwissCovid», was one of the first to be launched in June 2020 and follows the DP-3T blueprint. In May 2021, SwissCovid had around 1.76 million active users, corresponding to 20.5% of the Swiss population [11]. Details on the design and implementation of SwissCovid are reported elsewhere [8, 12].

DPT has three potential advantages over MCT [12]. First, the notification of exposed DPT app users is automatized once the index case has triggered the notification, leading to a potential speed advantage over MCT in interrupting transmission chains. Second, DPT still functions when MCT is at capacity due to high case numbers. Third, DPT has a wider reach than MCT because it does not rely on the infected individual’s recollection of his or her encounters. However, DPT apps are complex interventions involving multiple, sequential steps and specific actions by app users to exert their effect (notification cascade [13], Supplementary File 1). To date, the possible impact of DPT apps on pandemic mitigation is only partially understood. Some modeling studies reported that DPT, alone or in combination with MCT, can have an effect in reducing SARS-CoV-2 transmission [7, 14, 15]. However, they relied on several strong assumptions, indicating that DPT effectiveness strongly depends on population uptake and the timeliness of case identification and quarantining of contacts [16, 17]. Population-level data from the Isle of Wight [18] and more recently from England and Wales [19] provided evidence of the impact of the NHS COVID-19 app on SARS-CoV-2 incidence. In the latter analysis, Wymant et al. estimated that app notifications averted between approximately 200,00–900,000 cases between November and December 2020 [19].

Only few empirical analyses on the effectiveness of SwissCovid exist. Two studies identified factors associated with DPT app uptake and reasons for non-use [20], as well as challenges related to the implementation of SwissCovid in Switzerland [21]. Salathé et al. analyzed publicly available performance indicators for SwissCovid, such as number of app downloads and notification codes (CovidCodes) entered, and demonstrated proof-of-principle for the functioning of the app [22]. Furthermore, findings from a recent simulation study of the SwissCovid notification cascade in the Canton of Zurich suggest that DPT notifications may have led to an additional 5% of exposed persons entering quarantine in September 2020 [13].

Yet, critical questions pertaining to other conditions necessary for the functioning of DPT apps and their real-world impact remain unanswered. In particular, it is unclear whether the app indeed reduces the time between exposure and entering quarantine in close contacts. Using data from the Zurich SARS-CoV-2 Cohort, this study had two main aims. First, we evaluated the adherence of SwissCovid app users with the recommended steps (i.e., uploading of CovidCodes by cases upon testing positive for SARS-CoV-2, and quarantine and testing by close contacts upon receiving an app notification). Second, we examined the effectiveness of SwissCovid by evaluating whether the time from exposure to quarantine differed between close contacts who have or have not received an app warning.

## Methods

### Study Design and Participants

The Zurich SARS-CoV-2 Cohort Study is an ongoing, prospective, longitudinal, population-based cohort study of individuals infected with SARS-CoV-2 and their close contacts in the Canton of Zurich. The cohort was established in collaboration with the Cantonal Health Directorate Zurich and aims to characterize clinical outcomes and immunological responses of index cases and examine patterns of transmission among index cases and their close contacts.

Individuals diagnosed with SARS-CoV-2 infection and their close contacts were identified through mandatory laboratory reporting of positive cases to and routine contact tracing by the Cantonal Health Directorate. All identified cases and close contacts were screened for eligibility and invited if they were ≥18 years old, residing in the Canton of Zurich, had sufficient knowledge of the German language and were able to follow the study procedures. Random sampling of the two populations was performed on a daily basis. Sampling of cases was stratified by age and close contacts were sampled in clusters based on the respective index case. Informed consent was obtained from all individuals agreeing to participate in the study. In this analysis, we used data from cases and close contacts enrolled between August 07, 2020 and September 30, 2020, when conditions changed due to a sharp increase in case numbers in Switzerland in early October 2020 [23].

The study protocol was approved by the ethics committee of the Canton of Zurich (BASEC 2020-01739) and prospectively registered on the International Standard Randomised Controlled Trial Number Registry (ISRCTN14990068).

### Data Collection

Data was collected and managed through the Research Electronic Data Capture (REDCap) system. Questionnaires for cases included questions on socio-demographics, comorbidities, details on the suspected transmission event, symptoms and disease burden. Similarly, questionnaires for close contacts elicited information regarding socio-demographics, symptoms, experiences with quarantine, and details on their contact with the case (e.g., exposure setting, timing). Both questionnaires included questions related to the use of the SwissCovid app, receipt and uploading of CovidCodes by cases, and app warnings received by close contacts (Supplementary File 2).

Data from both questionnaires was available for ten individuals who were initially enrolled as close contacts and later tested SARS-CoV-2 positive. Individuals identified as close contacts by contact tracing who tested positive before study enrollment were directly enrolled as cases and thus provided data from only one questionnaire (n = 55; Supplementary File 3). Individuals pertaining to either group were considered “converted cases” in the analysis and contributed data on the level of close contacts and cases, as appropriate.

### Definitions

Participants reporting permanent or occasional use of SwissCovid were considered app users. Self-reported exposure settings were classified as household if the participant reported living in the same household as the case. Non-household settings included workplace, private settings, public settings, healthcare facility, school or university, shared accommodation, and military. In line with the definition by the Cantonal Health Directorate, the exposure date referred to the last day when the close contact was within 1.5 m distance of the case for ≥15 min up to 48 h before symptom onset (or positive test if asymptomatic) and without personal protective equipment. For household contacts, exposure date corresponded to the first day the case was isolated. Exposure dates were recorded by two methods: self-reported by participants (main measure) and a proxy measure defined as 10 days prior to the last day of quarantine, as determined by contact tracing.

### Outcomes

To evaluate adherence, primary outcomes included the frequency of cases who received and uploaded the CovidCode (thereby triggering a warning of contacts), frequency of close contacts who received a SwissCovid app notification and among those, and the frequency of close contacts who received the notification before being contacted by MCT. Regarding effectiveness, our primary outcome was the time interval (in days) between exposure date and the beginning of quarantine among close contacts, comparing those notified by the app to those not notified by the app.

### Statistical Methods

Adherence with recommended actions was evaluated using descriptive statistics. Continuous variables are presented as median and interquartile ranges (IQR) and categorical variables as frequencies (N) and percentages (%). Free text responses regarding reasons for non-use of the app, not uploading the CovidCode by cases and steps taken by close contacts after receiving a warning, were reviewed. Based on their context, responses were coded without a preconceived categorization and reported in frequency and percentage.

To evaluate effectiveness (i.e., time from exposure to beginning of quarantine), close contacts were grouped into “app notified” and “not app notified.” App non-users were considered “not app notified.” We assumed that notification time in household settings would be intrinsically faster than in non-household settings due to differences in information pathways and thus stratified participants by exposure setting (household vs. non-household). Concordance between self-reported and proxy exposure dates was examined. If self-reported date of last exposure was later than the beginning of quarantine (e.g., in same household contacts where the exposure date was not clearly defined), we used the proxy exposure date. If contacts entered quarantine on the day of exposure (leading to a 0 day interval) a delay of 0.5 days was added. Differences between groups were explored using Kaplan-Meier curves and stratified log-rank test. To evaluate the association between app notification and time from exposure to quarantine, we used a Cox proportional hazards model stratified by exposure setting and adjusted for age group, sex, education and employment status. Non-proportionality and possible influential outliers were tested using the scaled Schoenfeld residuals and *dfbeta* values, respectively. The model was adjusted for the cluster effect of sampling using robust variance estimation. Hazard ratios (HRs) and 95% confidence intervals (CI) were reported. We explored the robustness of our findings by performing a sensitivity analysis using the proxy exposure date instead of the self-reported exposure date to estimate the time from exposure to quarantine. Furthermore, in a second sensitivity analysis, we restricted our analysis to those using the app to account for potential confounding mediated by app use and associated characteristics of the close contacts. All analyses were performed using R version 3.6.1.

### Role of the Funding Source

Study funders had no role in the study design, data collection, analysis, interpretation, or writing of this report. All authors had access to the data in the study and accept responsibility to submit for publication.

## Results

### Study Population

Between August 06, 2020 and October 01, 2020, 2,519 individuals were diagnosed with SARS-CoV-2 in the Canton of Zurich and 6,316 close contacts were traced by contact tracing. Among cases, contact information and consent to be recontacted was available for 2082 individuals, among which 1,134 were eligible and invited to participate in our study. 392 cases agreed to participate (participation rate 35%), of which 65 cases had converted after being originally traced as a close contact. Among all close contacts, contact information was available for 5,545 individuals. 1,808 met our eligibility criteria, of which 734 were contacts of invited cases. 640 close contacts were invited and 271 individuals (261 close contacts and 10 converted cases) agreed to participate (participation rate 42%) (Supplementary File 3).

In this analysis, we thus included 328 cases, 65 cases that converted from originally being traced as a close contact and 261 close contacts. Cases and close contacts were largely similar with respect to socio-demographic characteristics (Table 1). Median age of cases and close contacts at time of identification was 38 and 35 years, respectively. Approximately 50% of the participants in both groups were female. Other characteristics such as Swiss nationality (79 and 84%), level of education (55 and 62% with a university or technical college degree), employment status (81 and 80% employed) and self-reported comorbidities (22 and 23% with at least one comorbidity) were also comparable between close contacts and cases. Converted cases were slightly different from the other two groups, with approximately 54% being female and 92% Swiss nationals.

**TABLE 1:** Baseline characteristics of the study population (data from the Zurich SARS-CoV-2 Cohort Study: 2020–2021, Switzerland).

Variable	Close contact, *N* = 261	Converted case, *N* = 65	Case, *N* = 328
Age, years^*^	35 (28, 51)	39 (29, 55)	38 (29, 51)
Sex			
Female	128/261 (49.0%)	35/65 (53.8%)	164/328 (50.0%)
Male	133/261 (51.0%)	30/65 (46.2%)	164/328 (50.0%)
Education			
Up to mandatory school	9/257 (3.5%)	4/65 (6.2%)	13/327 (4.0%)
Vocational training/specialized baccalaureate	89/257 (34.6%)	22/65 (33.8%)	133/327 (40.7%)
Technical college or university studies	159/257 (61.9%)	39/65 (60%)	181/327 (55.3%)
(Missing)	4	0	1
Employment status			
Employed	205/256 (80.1%)	53/64 (82.8%)	265/327 (81.0%)
Student	33/256 (12.9%)	6/64 (9.4%)	27/327 (8.3%)
Unemployed	18/256 (7.0%)	5/64 (7.8%)	35/327 (10.7%)
(Missing)	5	1	1
Monthly household income (Swiss Francs)			
<6,000	87/247 (35.2%)	21/62 (33.9%)	111/314 (35.4%)
6,000–12,000	101/247 (40.9%)	32/62 (51.6%)	120/314 (38.2%)
>12,000	59/247 (24.9%)	9/62 (14.5%)	83/314 (26.4%)
(Missing)	14	3	14
Nationality			
Swiss	220/261 (84.3%)	60/65 (92.3%)	260/328 (79.3%)
Non-Swiss	41/261 (15.7%)	5/65 (7.7%)	68/328 (20.7%)
Chronic comorbid conditions			
At least one self-reported comorbid chronic condition	57/252 (23%)	13/61 (21.3%)	69/318 (21.7%)
No self-reported chronic comorbid conditions	195/252 (77%)	48/61 (78.7%)	249/318 (78.3%)
(Missing)	9	4	10
Known exposure setting			
Knows or has strong suspicion	253/257 (98.4%)	60/65 (92.3%)	152/328 (46.3%)
No	4/257 (1.6%)	5/65 (7.7%%)	176/328 (53.7%)
(Missing)	4	0	0
Exposure setting (among those with known/suspected exposure)			
Household	72/252 (28.6%)	32/60 (53.4%)	22/150 (14.6%)
Workplace	43/252 (17.1%)	2/60 (3.3%)	23/150 (15.3%)
Private setting	66/252 (26.2%)	15/60 (25.0%)	42/150 (28.0%)
Public space	45/252 (17.9%)	8/60 (13.3%)	43/150 (28.7%)
School/University	8/252 (3.2%)	0/60 (0.0%)	1/150 (0.7%)
Other	18/252 (7.0%)	3/60 (5.0%)	19/150 (12.7%)
Healthcare facility	0/252 (0.0%)	0/60 (0.0%)	0/150 (0.0%)
(Missing)	9	5	178
SwissCovid app use			
App non-user	73/258 (28.3%)	22/64 (34.4%)	125/326 (38.3%)
App user	185/258 (71.7%)	42/64 (65.6%)	201/326 (61.7%)
(Missing)	3	1	2
Reasons for non-use of the app			
No knowledge of the app	1/57 (1.8%)	2/16 (12.5%)	5/104 (4.8%)
Perception of uselessness	13/57 (22.8%)	3/16 (18.8%)	29/104 (27.9%)
Technical problems	14/57 (24.5%)	4/16 (25.0%)	21/104 (20.2%)
Privacy and data protection	16/57 (28.1%)	5/16 (31.2%)	23/104 (22.1%)
Other	13/57 (22.8%)	2/16 (12.5%)	26/104 (25.0%)
(Missing)	16	6	21

* Median (IQR).

The exposure setting was reported as known or strongly suspected by 98% of close contacts and 93% of converted cases. Meanwhile, only 46% of cases knew or suspected the setting in which SARS-CoV-2 transmission occurred. Among those with knowledge or suspicion regarding their exposure, household and private settings were most frequently reported among close contacts (29 and 26%) and converted cases (53 and 26%). Cases most frequently stated public spaces (29%) and private settings (28%) as the exposure setting.

### Adherence

Overall, 62% (n = 201) of cases, 66% (n = 42) of converted participants, and 72% (n = 185) of close contacts were app users. Reasons for app non-use are reported in Table 1 and Supplementary File 4. On average, app non-users were older and a higher proportion were female, retired and non-Swiss nationals compared to app users (Supplementary File 5).

Among 243 cases using SwissCovid, 92% (n = 224) reported to have received a CovidCode from public health authorities. Of those, 96% (n = 215) uploaded the code in the app, thus triggering a notification to potentially exposed contacts. Main reasons for not uploading the code included receiving it too late or that their close contacts were already in quarantine (Table 2).

**TABLE 2:** CovidCodes received and uploaded by cases who are app users (data from the Zurich SARS-CoV-2 Cohort Study: 2020–2021, Switzerland).

Variable	*N* = 243^*^
Received CovidCode	224/243 (92.2%)
Uploaded CovidCode	215/224 (96.0%)
Reasons for not uploading the CovidCode	
Did not work/Code invalid	3/8 (37.5%)
Received the code too late/Had already informed their contacts	2/8 (25.0%)
Believed data has already been deleted	1/8 (12.5%)
Close contacts already in quarantine	1/8 (12.5%)
Not yet received	1/8 (12.5%)
(Missing)	1

* Includes data from 42 converted cases.

Among the 192 close contacts using the app, 38% (n = 73) received an app notification within 7 days of the last relevant exposure. 43 of these reported a non-household exposure setting, corresponding to 34% of all non-household app users. Out of all contacts receiving a notification, 12% (n = 9) received the notification before being contacted by MCT. After receiving the app notification, 14% of the 73 close contacts followed the recommendation of calling the SwissCovid info-line, whilst the remainder undertook other (19%) or no actions (67%). Most participants taking no action stated that they had already been reached by MCT and were already in quarantine and/or tested for SARS-CoV-2 (Table 3).

**TABLE 3:** App notifications received and steps taken after by close contacts who are app users (data from the Zurich SARS-CoV-2 Cohort Study: 2020–2021, Switzerland).

Variable	Close contacts; *N* = 192^a^
Received a notification by the app	
Yes, in the last 7 days probably because of the current contact	73/192 (38.0%)
Yes, more than 7 days ago	2/192 (1.0%)
No notification	117/192 (60.9%)
Notified by the app before the cantonal medical service	9/73 (12.3%)
(Missing)	2
Steps taken after receiving an app notification	
Called SwissCovid infoline	10/72 (13.9%)
Other steps taken	14/72 (19.4%)
No steps taken	48/72 (66.7%)
(Missing)	3
Other steps taken	
Had already taken measure^b^ after being traced by contact tracing	6/14 (42.9%)
Had already taken measures^b^ following family/friend’s advice	6/14 (42.9%)
Called cantonal medical service	1/14 (7.1%)
Testing	1/14 (7.1%)

a Includes 7 “Converted” cases for whom data as a close contact was available (i.e. converted after enrolment).

b Includes SARS-CoV-2 testing and quarantine.

### Effectiveness

The median time from last exposure to beginning of quarantine among all close contacts was 2 days (IQR 1–3 days) based on the self-reported exposure date (main analysis). When using the proxy exposure date, the median time to quarantine was 1 day (IQR 0.5–3 days, sensitivity analysis). There was a 69% concordance between self-reported and proxy exposure date. 20 close contacts reported to have had the last exposure after starting quarantine, 18 of which reported the case to be a household member and two a friend.

**FIGURE 1:**
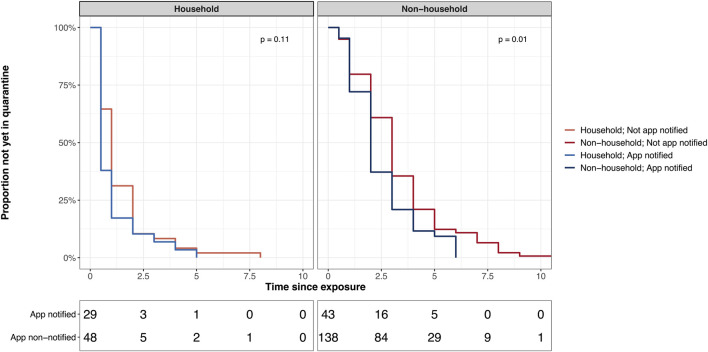
Time from exposure to quarantine in app notified versus not app notified stratified by exposure setting. Figure includes only participants from whom data on the beginning of quarantine is available. Data from the Zurich SARS-CoV-2 Cohort Study: 2020–2021, Switzerland.

We found that the time from exposure to quarantine differed across exposure settings and between contacts that received or did not receive an app notification (Figure 1). Overall, household contacts had a shorter median time from exposure to quarantine than non-household contacts (1 vs. 3 days). In *non-household* settings, we found a difference in time intervals indicating a shorter duration to quarantine in app notified (n = 43; median 2 days, IQR 1–3) compared to non-app notified contacts [n = 138 (missing data on time interval from four people); median 3 days, IQR 2–4; *p* = 0.01]. We found similar results after excluding non-app users (Supplementary File 6). Among the 43 app notified non-household contacts, 8 (18.6%) reported to have received the app notification before they were contacted by MCT. 47% of app notified non-household contacts reported to have decided themselves to initiate quarantine compared to 31% of non-app notified non-household contacts. In app notified contacts that received the warning before MCT, 75% (6/8) reported self-quarantine as the initial reason for quarantine, compared to 40% (14/35) of those receiving the warning after MCT (Supplementary File 7). However, in *household* settings, there was no evidence for a difference in the time from exposure to quarantine between app notified (median 0.5 days, IQR 0.5–2.0) and non-app notified contacts (median 1 day, IQR 0.5–2.0; *p* = 0.11).

In the stratified multivariable Cox model, we found strong evidence that contacts notified by the app had a greater probability of going into quarantine earlier than those not notified by the app while adjusting for age, sex, education, and employment status (HR 1.53, 95% CI 1.15–2.03; *p* = 0.004). Age, education level, and employment status were not associated with a shorter time to quarantine (Table 4, Supplementary File 8). There was no evidence for an interaction between app notification and exposure setting. Sensitivity analyses using the proxy exposure date, as well as when restricting the analysis to app users, yielded similar results (Supplementary Files 9, 10).

**TABLE 4:** Multivariable cox proportional hazards analysis of time from exposure to quarantine in close contacts, stratified by exposure setting (data from the Zurich SARS-CoV-2 Cohort Study: 2020–2021, Switzerland).

Variable	AHR (95% CI)^*^	*p*-value
Age at diagnosis, years		
18–39	1 (reference)	—
40–64	1.28 (0·91–1.80)	0.16
65+	1.45 (0·67–3.12)	0.35
Sex		
Female	1 (reference)	—
Male	0.86 (0.63–1.15)	0.31
Highest education level		
Mandatory school	1 (reference)	—
Vocational training/baccalaureate	0.67 (0.34–1.32)	0.24
Technical college or university	0.61 (0.29–1.27)	0.19
Employment status		
Employed	1 (reference)	—
Student	1.41 (0.91–2.18)	0.12
Unemployed	0.85 (0.38–1.93)	0.70
App notification		
App non-notified	1 (reference)	—
App notified	1.53 (1.15–2.03)	0.004

AHR: adjusted hazard ratio, CI: confidence interval.

* Model adjusted for age group, sex, highest education level, employment status and app notification.

## Discussion

In this study of 261 close contacts and 393 cases (including 65 converted individuals) identified through routine contact tracing in the Canton of Zurich, we evaluated use of SwissCovid and whether it provides a time advantage over MCT. Our analysis showed that non-household contacts notified by the app started quarantine earlier than those not notified by the app. This provides important evidence that DPT apps have an impact on the timely interruption of transmissions chains.

Most household contacts entered quarantine the same or the following day after exposure to a case in our study. This was expected, as they are easier to contact and are commonly informed directly by the case about their exposure. On the other hand, the contacting of non-household contacts through MCT is often more time-consuming and longer delays may be expected. We found evidence for a possible time advantage through the app in non-household setting, with app-notified contacts entering quarantine on average 1 day earlier than those not notified by the app. Considering that the testing delay (i.e., time from symptom onset to positive test) is 2.5 days on average in the Canton of Zurich [24], tracing delays and an overall reduced effectiveness of a contact tracing strategy are to be expected [14]. However, this does not explain differences between app notified and not app notified contacts. To explain this difference, we descriptively explored multiple hypotheses. We found that a higher percentage of app notified non-household contacts reported to have entered self-quarantine compared to those not notified by the app (47 vs. 31%). This finding supports the hypothesis that receiving an app notification may lead to a shorter time between exposure and quarantine. Although only 8 (19%) of 43 app notified contacts received the app warning before being reached by MCT, app notifications received after being called by contact tracers may not be without effect. For example, these notifications could have a reinforcing effect on the quarantine recommendations by MCT. We additionally explored alternative hypotheses that could explain our findings, such as confounding by case characteristics (e.g., more symptomatic cases or earlier testing among app users). However, we found no indication for systematic confounding in our descriptive analyses. Additionally, as the non-app notified group in our main analysis also included non-app users, our finding of a difference in time to quarantine may have potentially been influenced by better compliance of app users. Nonetheless, we found similar results after restricting our analysis to only app users.

Our findings constitute the first evidence that DPT may be effective in reaching close contacts faster than MCT. Albeit small, such a time difference may be relevant in reducing transmission in the population. Ferretti et al. demonstrated in a modeling study that reducing the time to quarantine from 3 to 2 days had a substantial impact on reducing the spread of SARS-CoV-2, assuming that a large fraction of the population is using the app [7]. This emphasizes the need to focus on behavioral aspects of app uptake and use for the implementation of DPT, as well as to consider specific subgroups in its evaluation, such as distinguishing between different exposure settings.

In our study, participants were enrolled during a period when case numbers were comparatively low. One potential advantage of the app is that it may be even more effective in times when case numbers are high leading to capacity issues in MCT. However, this requires that app coverage is sufficient and an efficient process is in place to initiate the notification cascade [13, 22]. Thus, our findings may underestimate the effectiveness of the SwissCovid app in situations where MCT is overwhelmed. In addition, this analysis is restricted to close contacts that were identified by MCT, due to the design of the Zurich SARS-CoV-2 Cohort study. However, another potential advantage of the app is in warning exposed individuals that were unknown to the case or about whom they had forgotten [7]. This setting is difficult to assess given the privacy-by-design principle implemented in the SwissCovid app and was not within the scope of this analysis. As a consequence, our analysis did not consider potential additional benefits arising in the context of such exposure events.

A high percentage of participants reported using the app, exceeding previous estimates based on publicly available data and other population-based surveys [20, 22]. This difference may be explained by participants enrolled in the Zurich SARS-CoV-2 Cohort study being better informed about COVID-19 and more compliant with preventive public health measures than non-participants. Based on our data, the generation and uploading of CovidCodes seemed efficient, which contrasts previous reports of an approximate 30% gap between generated and entered CovidCodes [22]. Nevertheless, some of the participants also reported significant delays in receiving the CovidCodes. Until recently, the SwissCovid notification cascade suffered a few bottlenecks relating to the CovidCode generation process. Prior to October 2020, issuance of the codes was linked to MCT. It relied on the contact tracers to clarify whether a case requires a code during the initial tracing call and relaying the information to another person responsible for issuing the codes. In parallel to the generation and sending of CovidCodes to the cases, the contact tracer would continue to call the contacts. As the app would take a few hours until it pushes the notifications, MCT would have in theory reached the close contact first. This may also be reflected in the relatively low proportion of close contacts being notified by the app before being reached by MCT. However, although most contacts were reached the same day, contact tracers sometimes had trouble reaching the contacts by phone, leading to time delays and providing the app with a time advantage over MCT. Additional steps have been taken to improve the efficiency of the processes necessary for reducing such delays. In October, the CovidCode generation process was separated from MCT and cases were able to personally request codes through text messages. Additionally, health service providers such as laboratories, pharmacies and testing centers were able to generate and issue CovidCodes to cases starting November 18, 2020 to ensure a more rapid initiation of the notification cascade. Since December 12, 2020, CovidCodes are generated and sent automatically through an online form completed by cases as the first step in MCT in the Canton of Zurich.

Some further limitations of our study should be noted. Selection effects during enrollment may have led to a generally more health literate or compliant study population. However, such self-selection effects would not invalidate the proof-of-principle of our analysis but limit the transportability of our findings to the general population. Furthermore, despite consistent signals in our data, a causality between app notification and faster quarantine could not be unequivocally demonstrated. But the observation of a small subgroup of contacts who received the app notification and entered quarantine before being reached by MCT instills confidence that SwissCovid, in principle, achieves one of its main goals. Further studies are needed to quantify the impact of our findings on pandemic mitigation.

To our knowledge, our study is the first to evaluate the real-world effectiveness of a DPT app and leverages data from a population-based cohort study. While a more in-depth assessment of the exact sequence and timing of events related to the notification cascade may shed further light on the impact of SwissCovid on an individual level, our findings confirm the hypothesized benefit of DPT apps alarming non-household contacts earlier than MCT, thereby leading to earlier quarantine.

## Data Availability

We are open to sharing individual participant data that underlie the results reported in this article, after de-identification upon reasonable requests to the corresponding author. Data requestors will need to sign a data access agreement.

## References

[1] World Health Organization. Contact Tracing in the Context of COVID-19. [Internet] (2020). Available from: https://www.who.int/publications-detail-redirect/contact-tracing-in-the-context-of-covid-19 (Accessed November 13, 2020).

[2] European Centre for Disease Prevention and Control. Guidelines for the Implementation of Non-pharmaceutical Interventions against COVID-19. [Internet]. European Centre for Disease Prevention and Control (2020). Available from: https://www.ecdc.europa.eu/en/publications-data/covid-19-guidelines-non-pharmaceutical-interventions (Accessed November 13, 2020).

[3] Bi Q Wu Y Mei S Ye C Zou X Zhang Z Epidemiology and Transmission of COVID-19 in 391 Cases and 1286 of Their Close Contacts in Shenzhen, China: a Retrospective Cohort Study. [Internet]. Lancet Infect Dis (2020) 911. Available from: https://www.thelancet.com/journals/laninf/article/PIIS1473-3099(20)30287-5/abstract. 10.1101/2020.03.03.20028423 (cited 2020 May 23) 32353347 PMC7185944

[4] Ng Y Li Z Chua YX Chaw WL Zhao Z Er B Evaluation of the Effectiveness of Surveillance and Containment Measures for the First 100 Patients with COVID-19 in Singapore - January 2-February 29, 2020. Morb Mortal Weekly Rep (2020) 69(11):307–11. 10.15585/mmwr.mm6911e1 PMC7739977 32191691

[5] Hellewell J Abbott S Gimma A Bosse NI Jarvis CI Russell TW Feasibility of Controlling COVID-19 Outbreaks by Isolation of Cases and Contacts. Lancet Glob Health (2020) 8(4):e488–e496. 10.1016/s2214-109x(20)30074-7 32119825 PMC7097845

[6] Peak CM Kahn R Grad YH Childs LM Li R Lipsitch M Individual Quarantine versus Active Monitoring of Contacts for the Mitigation of COVID-19: a Modelling Study. Lancet Infect Dis (2020) 20(9):1025–33. 10.1016/s1473-3099(20)30361-3 32445710 PMC7239635

[7] Ferretti L Wymant C Kendall M Zhao L Nurtay A Abeler-Dörner L Quantifying SARS-CoV-2 Transmission Suggests Epidemic Control with Digital Contact Tracing. [Internet]. Science (2020) 368(6491):eabb6936. 10.1126/science.abb6936 32234805 PMC7164555

[8] Swiss National Covid-19 Science Task Force. Digital Proximity Tracing. Policy Briefs. [Internet]. Available from: https://ncs-tf.ch/de/policy-briefs/digital-proximity-tracing-15-may-20-en/download (Accessed November 22, 2020).

[9] Troncoso C Payer M Hubaux J-P Salathé M Larus J Bugnion E Decentralized Privacy-Preserving Proximity Tracing. [cs] [Internet]. arXiv:200512273 (2020). Available from: http://arxiv.org/abs/2005.12273 (Accessed May 25, 2020).

[10] Covid Watch Whitepaper. Using Crowdsourced Data to Slow Virus Spread. [Internet]. Available from: https://blog.covidwatch.org/en/covid-watch-whitepaper-using-crowdsourced-data-to-slow-virus-spread (Accessed May 7, 2021).

[11] SwissCovid-App-Monitoring [Internet]. Available from: https://www.experimental.bfs.admin.ch/expstat/de/home/innovative-methoden/swisscovid-app-monitoring.html (Accessed November 22, 2020)

[12] von Wyl V Bonhoeffer S Bugnion E Puhan MA Salathé M Stadler T A Research Agenda for Digital Proximity Tracing Apps. [Internet]. Swiss Med Weekly (2020) 150:w20324. Available from: https://smw.ch/article/doi/smw.2020.20324. 10.4414/smw.2020.20324 (cited 2020 Nov 22) 32672340

[13] Menges D Aschmann HE Moser A Althaus CL von Wyl V. A Data-Driven Simulation of the Exposure Notification Cascade for Digital Contact Tracing of SARS-CoV-2 in Zurich, Switzerland. JAMA Netw Open (2021) 4(4):e218184. 10.1001/jamanetworkopen.2021.8184 33929521 PMC8087953

[14] Kretzschmar ME Rozhnova G Bootsma MCJ van Boven M van de Wijgert JHHM Bonten MJM. Impact of Delays on Effectiveness of Contact Tracing Strategies for COVID-19: a Modelling Study. Lancet Public Health (2020) 5(8):e452–e459. 10.1016/s2468-2667(20)30157-2 32682487 PMC7365652

[15] Nuzzo A Tan CO Raskar R DeSimone DC Kapa S Gupta R. Universal Shelter-In-Place versus Advanced Automated Contact Tracing and Targeted Isolation. Mayo Clinic Proc (2020) 95(9):1898–905. 10.1016/j.mayocp.2020.06.027 PMC7306713 32861334

[16] Braithwaite I Callender T Bullock M Aldridge RW. Automated and Partly Automated Contact Tracing: a Systematic Review to Inform the Control of COVID-19. Lancet Digit Health (2020) 2(11):e607–e621. 10.1016/s2589-7500(20)30184-9 32839755 PMC7438082

[17] Anglemyer A Moore TH Parker L Chambers T Grady A Chiu K Digital Contact Tracing Technologies in Epidemics: a Rapid Review. [Internet]. Cochrane Database Syst Rev (2020) 8:CD013699. 10.1002/14651858.CD013699 33502000 PMC8241885

[18] Kendall M Milsom L Abeler-Dörner L Wymant C Ferretti L Briers M Epidemiological Changes on the Isle of Wight after the Launch of the NHS Test and Trace Programme: a Preliminary Analysis. Lancet Digit Health (2020) 2(12):e658–e666. 10.1016/s2589-7500(20)30241-7 33078140 PMC7556784

[19] Wymant C Ferretti L Tsallis D Charalambides M Abeler-Dörner L Bonsall D The Epidemiological Impact of the NHS COVID-19 App. Nature (2021) 594:408–12. 10.1038/s41586-021-03606-z 33979832

[20] von Wyl V Höglinger M Sieber C Kaufmann M Moser A Serra-Burriel M Drivers of Acceptance of COVID-19 Proximity Tracing Apps in Switzerland: Panel Survey Analysis. JMIR Public Health Surveill (2021) 7(1):e25701. 10.2196/25701 33326411 PMC7790736

[21] von Wyl V. Challenges for Nontechnical Implementation of Digital Proximity Tracing During the COVID-19 Pandemic: Media Analysis of the SwissCovid App. JMIR Mhealth Uhealth (2021) 9(2):e25345. 10.2196/25345 33606658 PMC7919847

[22] Salathé M Althaus C Anderegg N Antonioli D Ballouz T Bugnon E Early Evidence of Effectiveness of Digital Contact Tracing for SARS-CoV-2 in Switzerland. [Internet]. Swiss Med Weekly (2020) 150(5152). Available from: https://smw.ch/article/doi/smw.2020.20457. 10.4414/smw.2020.20457 (cited 2020 Dec 17) 33327003

[23] COVID-19 Switzerland. Coronavirus. [Internet]. Available from: https://www.covid19.admin.ch/en/overview (Accessed December 03, 2020).

[24] Regierungsrates des Kantons Zürich. Antwort RR 320/2020 Tracing oder Virus – wer hat die Nase vorn?. [Internet] (2020). Available from: https://kantonsrat-zh.talus.ch/de/politik/cdws/dok_geschaeft.php?did=6c3aad19f6234e889b7dc0669c215130-332&v=1&r=PDF&filename=Antwort_RR_320-2020_Tracing_oder_Virus__wer_hat_die_Nase_vorn&typ=pdf (Accessed December 14, 2020).

